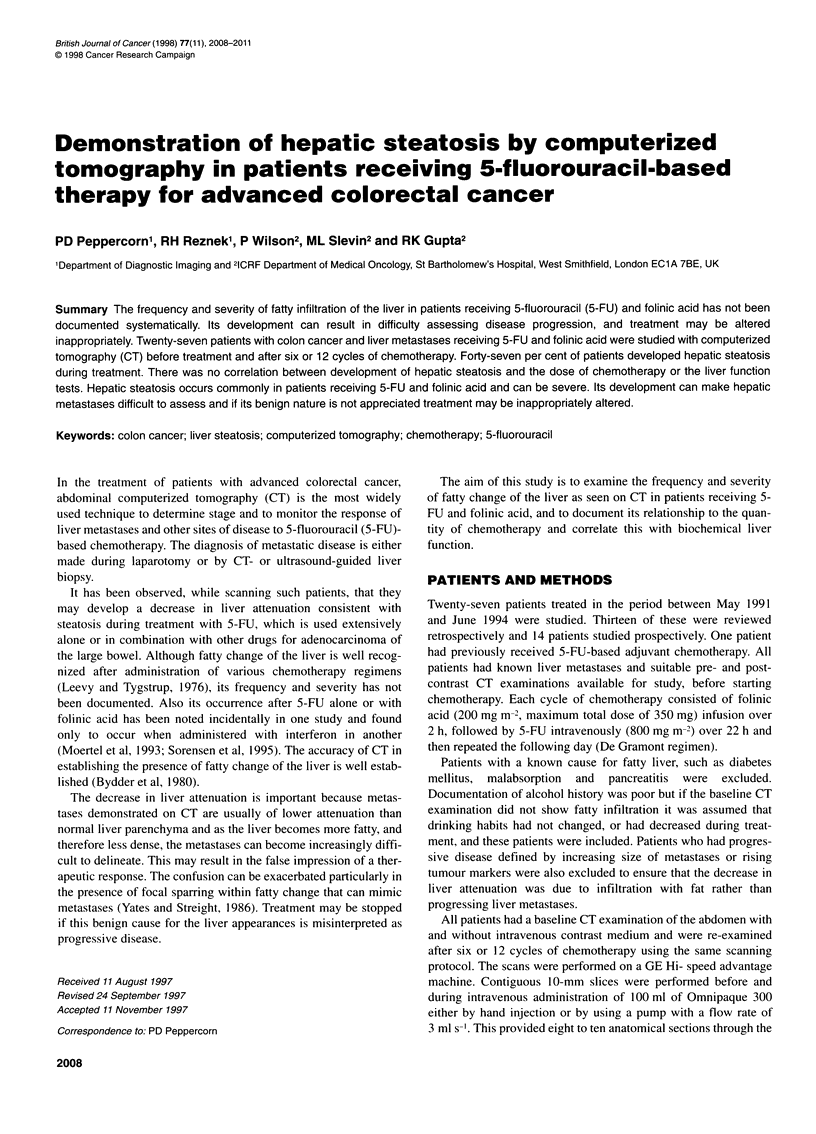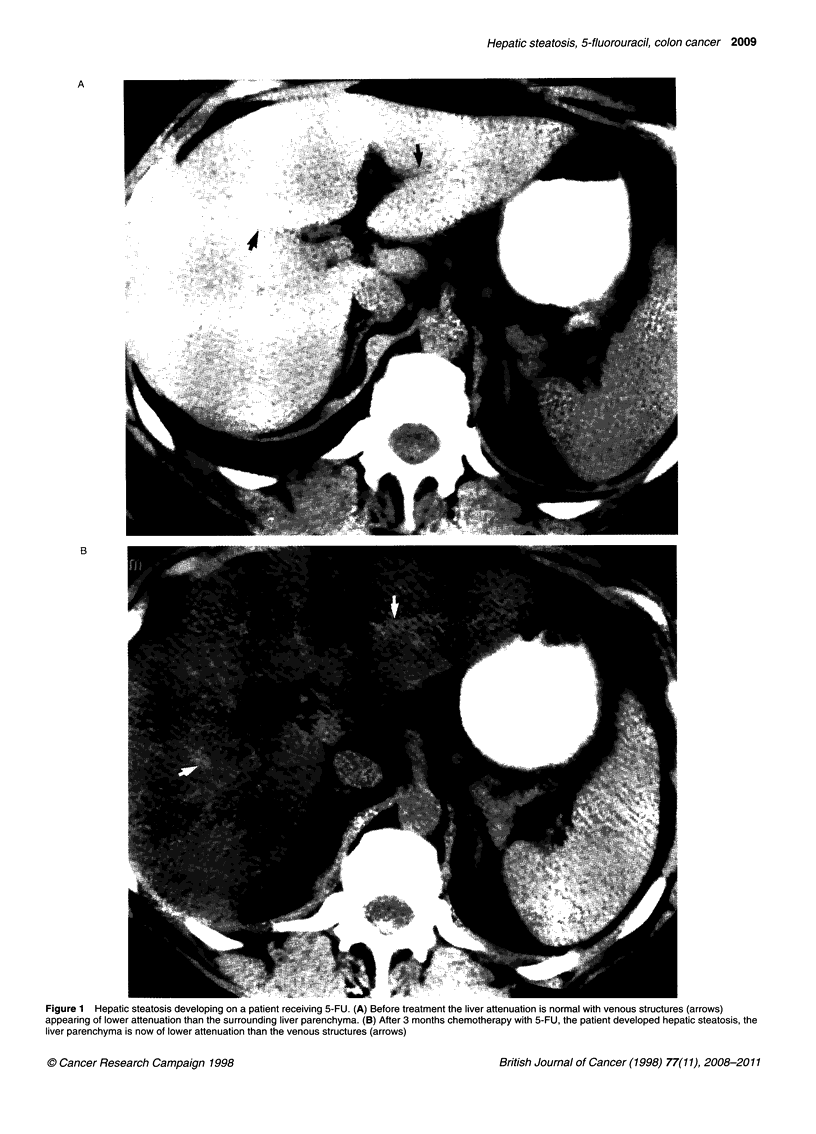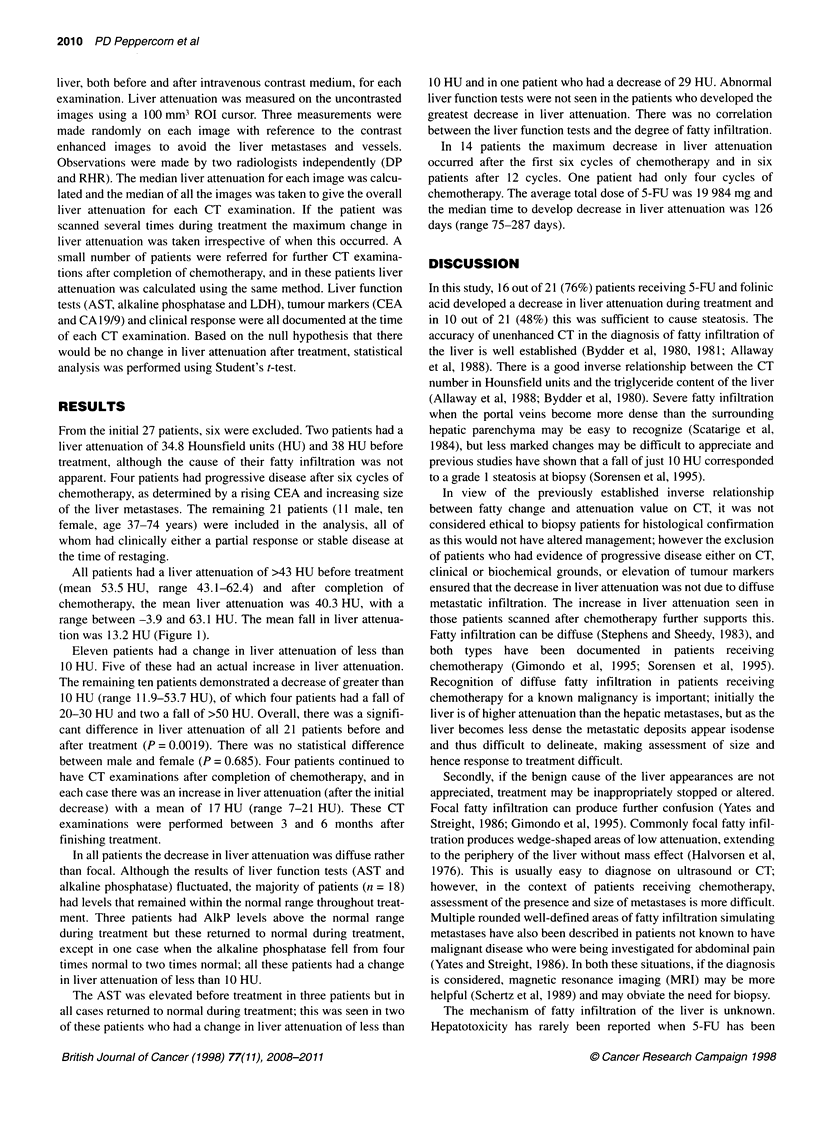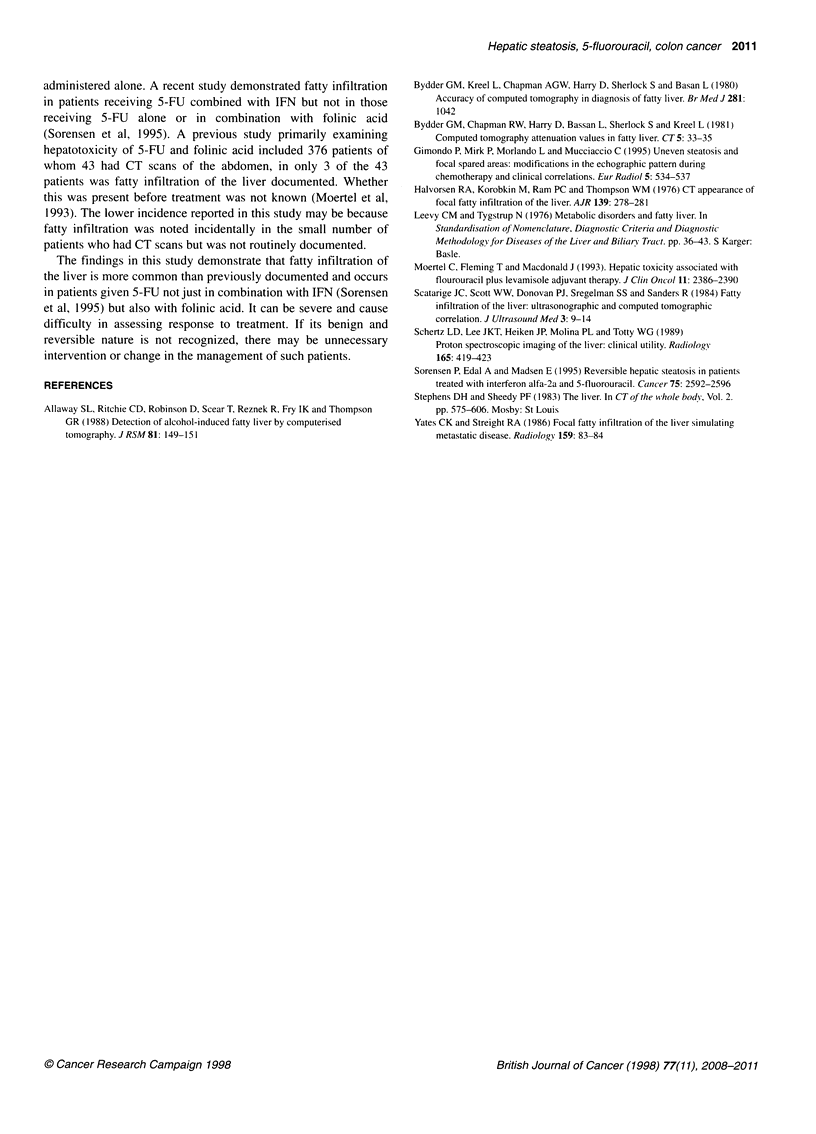# Demonstration of hepatic steatosis by computerized tomography in patients receiving 5-fluorouracil-based therapy for advanced colorectal cancer.

**DOI:** 10.1038/bjc.1998.333

**Published:** 1998-06

**Authors:** P. D. Peppercorn, R. H. Reznek, P. Wilson, M. L. Slevin, R. K. Gupta

**Affiliations:** Department of Diagnostic Imaging, St Bartholomew's Hospital, West Smithfield, London, UK.

## Abstract

**Images:**


British Journal of Cancer (1998) 77(11), 2008–2011
© 1998 Cancer Research Campaign

### Demonstration of hepatic steatosis by computerized tomography in patients receiving 5-fluorouracil-based therapy for advanced colorectal cancer

**PD Peppercorn[1], RH Reznek[1], P Wilson[2], ML Slevin[2] and RK Gupta[2]**

[1]Department of Diagnostic Imaging and [2]ICRF Department of Medical Oncology, St Bartholomew's Hospital, West Smithfield, London EC1A 7BE, UK

**Summary** The frequency and severity of fatty infiltration of the liver in patients receiving 5-fluorouracil (5-FU) and folinic acid has not been documented systematically. Its development can result in difficulty assessing disease progression, and treatment may be altered inappropriately. Twenty-seven patients with colon cancer and liver metastases receiving 5-FU and folinic acid were studied with computerized tomography (CT) before treatment and after six or 12 cycles of chemotherapy. Forty-seven per cent of patients developed hepatic steatosis during treatment. There was no correlation between development of hepatic steatosis and the dose of chemotherapy or the liver function tests. Hepatic steatosis occurs commonly in patients receiving 5-FU and folinic acid and can be severe. Its development can make hepatic metastases difficult to assess and if its benign nature is not appreciated treatment may be inappropriately altered.

**Keywords:** colon cancer; liver steatosis; computerized tomography; chemotherapy; 5-fluorouracil

In the treatment of patients with advanced colorectal cancer, abdominal computerized tomography (CT) is the most widely used technique to determine stage and to monitor the response of liver metastases and other sites of disease to 5-fluorouracil (5-FU)-based chemotherapy. The diagnosis of metastatic disease is either made during laparotomy or by CT- or ultrasound-guided liver biopsy.

It has been observed, while scanning such patients, that they may develop a decrease in liver attenuation consistent with steatosis during treatment with 5-FU, which is used extensively alone or in combination with other drugs for adenocarcinoma of the large bowel. Although fatty change of the liver is well recognized after administration of various chemotherapy regimens (Leevy and Tygstrup, 1976), its frequency and severity has not been documented. Also its occurrence after 5-FU alone or with folinic acid has been noted incidentally in one study and found only to occur when administered with interferon in another (Moertel et al, 1993; Sorensen et al, 1995). The accuracy of CT in establishing the presence of fatty change of the liver is well established (Bydder et al, 1980).

The decrease in liver attenuation is important because metastases demonstrated on CT are usually of lower attenuation than normal liver parenchyma and as the liver becomes more fatty, and therefore less dense, the metastases can become increasingly difficult to delineate. This may result in the false impression of a therapeutic response. The confusion can be exacerbated particularly in the presence of focal sparring within fatty change that can mimic metastases (Yates and Streight, 1986). Treatment may be stopped if this benign cause for the liver appearances is misinterpreted as progressive disease.

*Received 11 August 1997*
*Revised 24 September 1997*
*Accepted 11 November 1997*

*Correspondence to:* PD Peppercorn

The aim of this study is to examine the frequency and severity of fatty change of the liver as seen on CT in patients receiving 5-FU and folinic acid, and to document its relationship to the quantity of chemotherapy and correlate this with biochemical liver function.

### PATIENTS AND METHODS

Twenty-seven patients treated in the period between May 1991 and June 1994 were studied. Thirteen of these were reviewed retrospectively and 14 patients studied prospectively. One patient had previously received 5-FU-based adjuvant chemotherapy. All patients had known liver metastases and suitable pre- and post-contrast CT examinations available for study, before starting chemotherapy. Each cycle of chemotherapy consisted of folinic acid (200 mg $m^{-2}$, maximum total dose of 350 mg) infusion over 2 h, followed by 5-FU intravenously (800 mg $m^{-2}$) over 22 h and then repeated the following day (De Gramont regimen).

Patients with a known cause for fatty liver, such as diabetes mellitus, malabsorption and pancreatitis were excluded. Documentation of alcohol history was poor but if the baseline CT examination did not show fatty infiltration it was assumed that drinking habits had not changed, or had decreased during treatment, and these patients were included. Patients who had progressive disease defined by increasing size of metastases or rising tumour markers were also excluded to ensure that the decrease in liver attenuation was due to infiltration with fat rather than progressing liver metastases.

All patients had a baseline CT examination of the abdomen with and without intravenous contrast medium and were re-examined after six or 12 cycles of chemotherapy using the same scanning protocol. The scans were performed on a GE Hi- speed advantage machine. Contiguous 10-mm slices were performed before and during intravenous administration of 100 ml of Omnipaque 300 either by hand injection or by using a pump with a flow rate of 3 ml $s^{-1}$. This provided eight to ten anatomical sections through the

A

B

**Figure 1** Hepatic steatosis developing on a patient receiving 5-FU. (**A**) Before treatment the liver attenuation is normal with venous structures (arrows) appearing of lower attenuation than the surrounding liver parenchyma. (**B**) After 3 months chemotherapy with 5-FU, the patient developed hepatic steatosis, the liver parenchyma is now of lower attenuation than the venous structures (arrows)

© Cancer Research Campaign 1998

liver, both before and after intravenous contrast medium, for each examination. Liver attenuation was measured on the uncontrasted images using a 100 $mm^3$ ROI cursor. Three measurements were made randomly on each image with reference to the contrast enhanced images to avoid the liver metastases and vessels. Observations were made by two radiologists independently (DP and RHR). The median liver attenuation for each image was calculated and the median of all the images was taken to give the overall liver attenuation for each CT examination. If the patient was scanned several times during treatment the maximum change in liver attenuation was taken irrespective of when this occurred. A small number of patients were referred for further CT examinations after completion of chemotherapy, and in these patients liver attenuation was calculated using the same method. Liver function tests (AST, alkaline phosphatase and LDH), tumour markers (CEA and CA19/9) and clinical response were all documented at the time of each CT examination. Based on the null hypothesis that there would be no change in liver attenuation after treatment, statistical analysis was performed using Student's *t*-test.

### RESULTS

From the initial 27 patients, six were excluded. Two patients had a liver attenuation of 34.8 Hounsfield units (HU) and 38 HU before treatment, although the cause of their fatty infiltration was not apparent. Four patients had progressive disease after six cycles of chemotherapy, as determined by a rising CEA and increasing size of the liver metastases. The remaining 21 patients (11 male, ten female, age 37–74 years) were included in the analysis, all of whom had clinically either a partial response or stable disease at the time of restaging.

All patients had a liver attenuation of >43 HU before treatment (mean 53.5 HU, range 43.1–62.4) and after completion of chemotherapy, the mean liver attenuation was 40.3 HU, with a range between –3.9 and 63.1 HU. The mean fall in liver attenuation was 13.2 HU (Figure 1).

Eleven patients had a change in liver attenuation of less than 10 HU. Five of these had an actual increase in liver attenuation. The remaining ten patients demonstrated a decrease of greater than 10 HU (range 11.9–53.7 HU), of which four patients had a fall of 20–30 HU and two a fall of >50 HU. Overall, there was a significant difference in liver attenuation of all 21 patients before and after treatment ($P = 0.0019$). There was no statistical difference between male and female ($P = 0.685$). Four patients continued to have CT examinations after completion of chemotherapy, and in each case there was an increase in liver attenuation (after the initial decrease) with a mean of 17 HU (range 7–21 HU). These CT examinations were performed between 3 and 6 months after finishing treatment.

In all patients the decrease in liver attenuation was diffuse rather than focal. Although the results of liver function tests (AST and alkaline phosphatase) fluctuated, the majority of patients ($n = 18$) had levels that remained within the normal range throughout treatment. Three patients had AlkP levels above the normal range during treatment but these returned to normal during treatment, except in one case when the alkaline phosphatase fell from four times normal to two times normal; all these patients had a change in liver attenuation of less than 10 HU.

The AST was elevated before treatment in three patients but in all cases returned to normal during treatment; this was seen in two of these patients who had a change in liver attenuation of less than 10 HU and in one patient who had a decrease of 29 HU. Abnormal liver function tests were not seen in the patients who developed the greatest decrease in liver attenuation. There was no correlation between the liver function tests and the degree of fatty infiltration.

In 14 patients the maximum decrease in liver attenuation occurred after the first six cycles of chemotherapy and in six patients after 12 cycles. One patient had only four cycles of chemotherapy. The average total dose of 5-FU was 19 984 mg and the median time to develop decrease in liver attenuation was 126 days (range 75–287 days).

### DISCUSSION

In this study, 16 out of 21 (76%) patients receiving 5-FU and folinic acid developed a decrease in liver attenuation during treatment and in 10 out of 21 (48%) this was sufficient to cause steatosis. The accuracy of unenhanced CT in the diagnosis of fatty infiltration of the liver is well established (Bydder et al, 1980, 1981; Allaway et al, 1988). There is a good inverse relationship between the CT number in Hounsfield units and the triglyceride content of the liver (Allaway et al, 1988; Bydder et al, 1980). Severe fatty infiltration when the portal veins become more dense than the surrounding hepatic parenchyma may be easy to recognize (Scatarige et al, 1984), but less marked changes may be difficult to appreciate and previous studies have shown that a fall of just 10 HU corresponded to a grade 1 steatosis at biopsy (Sorensen et al, 1995).

In view of the previously established inverse relationship between fatty change and attenuation value on CT, it was not considered ethical to biopsy patients for histological confirmation as this would not have altered management; however the exclusion of patients who had evidence of progressive disease either on CT, clinical or biochemical grounds, or elevation of tumour markers ensured that the decrease in liver attenuation was not due to diffuse metastatic infiltration. The increase in liver attenuation seen in those patients scanned after chemotherapy further supports this. Fatty infiltration can be diffuse (Stephens and Sheedy, 1983), and both types have been documented in patients receiving chemotherapy (Gimondo et al, 1995; Sorensen et al, 1995). Recognition of diffuse fatty infiltration in patients receiving chemotherapy for a known malignancy is important; initially the liver is of higher attenuation than the hepatic metastases, but as the liver becomes less dense the metastatic deposits appear isodense and thus difficult to delineate, making assessment of size and hence response to treatment difficult.

Secondly, if the benign cause of the liver appearances are not appreciated, treatment may be inappropriately stopped or altered. Focal fatty infiltration can produce further confusion (Yates and Streight, 1986; Gimondo et al, 1995). Commonly focal fatty infiltration produces wedge-shaped areas of low attenuation, extending to the periphery of the liver without mass effect (Halvorsen et al, 1976). This is usually easy to diagnose on ultrasound or CT; however, in the context of patients receiving chemotherapy, assessment of the presence and size of metastases is more difficult. Multiple rounded well-defined areas of fatty infiltration simulating metastases have also been described in patients not known to have malignant disease who were being investigated for abdominal pain (Yates and Streight, 1986). In both these situations, if the diagnosis is considered, magnetic resonance imaging (MRI) may be more helpful (Schertz et al, 1989) and may obviate the need for biopsy.

The mechanism of fatty infiltration of the liver is unknown. Hepatotoxicity has rarely been reported when 5-FU has been

© Cancer Research Campaign 1998

administered alone. A recent study demonstrated fatty infiltration in patients receiving 5-FU combined with IFN but not in those receiving 5-FU alone or in combination with folinic acid (Sorensen et al, 1995). A previous study primarily examining hepatotoxicity of 5-FU and folinic acid included 376 patients of whom 43 had CT scans of the abdomen, in only 3 of the 43 patients was fatty infiltration of the liver documented. Whether this was present before treatment was not known (Moertel et al, 1993). The lower incidence reported in this study may be because fatty infiltration was noted incidentally in the small number of patients who had CT scans but was not routinely documented.

The findings in this study demonstrate that fatty infiltration of the liver is more common than previously documented and occurs in patients given 5-FU not just in combination with IFN (Sorensen et al, 1995) but also with folinic acid. It can be severe and cause difficulty in assessing response to treatment. If its benign and reversible nature is not recognized, there may be unnecessary intervention or change in the management of such patients.

### REFERENCES

Allaway SL, Ritchie CD, Robinson D, Scear T, Reznek R, Fry IK and Thompson GR (1988) Detection of alcohol-induced fatty liver by computerised tomography. *J RSM* **81**: 149–151

Bydder GM, Kreel L, Chapman AGW, Harry D, Sherlock S and Basan L (1980) Accuracy of computed tomography in diagnosis of fatty liver. *Br Med J* **281**: 1042

Bydder GM, Chapman RW, Harry D, Bassan L, Sherlock S and Kreel L (1981) Computed tomography attenuation values in fatty liver. *CT* **5**: 33–35

Gimondo P, Mirk P, Morlando L and Mucciaccio C (1995) Uneven steatosis and focal spared areas: modifications in the echographic pattern during chemotherapy and clinical correlations. *Eur Radiol* **5**: 534–537

Halvorsen RA, Korobkin M, Ram PC and Thompson WM (1976) CT appearance of focal fatty infiltration of the liver. *AJR* **139**: 278–281

Leevy CM and Tygstrup N (1976) Metabolic disorders and fatty liver. In *Standardisation of Nomenclature, Diagnostic Criteria and Diagnostic Methodology for Diseases of the Liver and Biliary Tract*. pp. 36–43. S Karger: Basle.

Moertel C, Fleming T and Macdonald J (1993). Hepatic toxicity associated with flourouracil plus levamisole adjuvant therapy. *J Clin Oncol* **11**: 2386–2390

Scatarige JC, Scott WW, Donovan PJ, Sregelman SS and Sanders R (1984) Fatty infiltration of the liver: ultrasonographic and computed tomographic correlation. *J Ultrasound Med* **3**: 9–14

Schertz LD, Lee JKT, Heiken JP, Molina PL and Totty WG (1989) Proton spectroscopic imaging of the liver: clinical utility. *Radiology* **165**: 419–423

Sorensen P, Edal A and Madsen E (1995) Reversible hepatic steatosis in patients treated with interferon alfa-2a and 5-fluorouracil. *Cancer* **75**: 2592–2596

Stephens DH and Sheedy PF (1983) The liver. In *CT of the whole body*, Vol. 2. pp. 575–606. Mosby: St Louis

Yates CK and Streight RA (1986) Focal fatty infiltration of the liver simulating metastatic disease. *Radiology* **159**: 83–84

© Cancer Research Campaign 1998